# Effect of acupuncture on Lipopolysaccharide-induced anxiety-like behavioral changes: involvement of serotonin system in dorsal Raphe nucleus

**DOI:** 10.1186/s12906-017-2039-y

**Published:** 2017-12-11

**Authors:** Tae Young Yang, Eun Young Jang, Yeonhee Ryu, Gyu Won Lee, Eun Byeol Lee, Suchan Chang, Jong Han Lee, Jin Suk Koo, Chae Ha Yang, Hee Young Kim

**Affiliations:** 1 College of Korean Medicine, Daegu Haany University, Daegu, 42158 South Korea; 2 Korean Medicine Fundamental Research Division, Korea Institute of Oriental Medicine, Daejeon, 34054 South Korea; 3 Department of Bioresource Science, Andong National University, Andong, 36729 South Korea; 4 Department of Physiology, College of Korean Medicine, Daegu Haany University, Daegu, 42158 South Korea

**Keywords:** Acupuncture, Anxiety, Dorsal raphe nucleus, LPS, Serotonin transporter

## Abstract

**Background:**

Acupuncture has been used as a common therapeutic tool in many disorders including anxiety and depression. Serotonin transporter (SERT) plays an important role in the pathology of anxiety and other mood disorders. The aim of this study was to evaluate the effects of acupuncture on lipopolysaccharide (LPS)-induced anxiety-like behaviors and SERT in the dorsal raphe nuclei (DRN).

**Methods:**

Rats were given acupuncture at ST41 (*Jiexi*), LI11 (*Quchi*) or SI3 (*Houxi*) acupoint in LPS-treated rats. Anxiety-like behaviors of elevated plus maze (EPM) and open field test (OFT) were measured and expressions of SERT and/or c-Fos were also examined in the DRN using immunohistochemistry.

**Results:**

The results showed that 1) acupuncture at ST41 acupoint, but neither LI11 nor SI3, significantly attenuated LPS-induced anxiety-like behaviors in EPM and OFT, 2) acupuncture at ST41 decreased SERT expression increased by LPS in the DRN.

**Conclusions:**

Our results suggest that acupuncture can ameliorate anxiety-like behaviors, possibly through regulation of SERT in the DRN.

## Background

Anxiety disorders, also called as generalized, social anxiety and panic disorder, are the most common mental health disorders which are characterized by irritability, fatigue, presence of restlessness, muscle tension, sleep problems, an intense and persistent fear of social, recurrent and unexpected panic attacks [1–3]. Anxiety disorders are also significantly related to other physiological dysfunctions such as migraine headaches, respiratory diseases, gastrointestinal diseases, and arthritis and can negatively affect mobility, social function, and health care [4].

Among neurotransmitters, serotonin (5-hydroxytryptamine or 5-HT) is critically involved in the pathophysiology of mood and anxiety disorders [5]. Several lines of evidence indicate that serotonin transporter (SERT or 5-HTT), responsible for high-affinity serotonin uptake from extracellular fluid at the synaptic cleft, plays important roles in the pathology of depression and other mood disorders [6–8]. A previous study showed that reduction of tryptophan hydroxylase (enzyme for serotonin synthesis) and SERT in ventromedial prefrontal cortex (vmPFC) and increased SERT in dorsal raphe nucleus (DRN) are associated with mood- and anxiety-like behavior in animal model [9]. In addition, the patients with anxiety disorder reveal enhanced serotonin synthesis [10] and reduced serotonin 1A receptor levels [11] in the amygdala and prescribe selective serotonin reuptake inhibitors (SSRIs) targeting SERT [12, 13].

Lipopolysaccharide (LPS), a bacterial endotoxin, causes physiological or psychiatric changes such as anhedonia, anorexia, depressed mood, apathy [14, 15] and inflammation linked to anxiety and depression [16]. LPS can trigger depressive symptoms in humans [14] and anxiety- and depressive-like behaviors in experimental animals [16, 17]. The underlying mechanism includes increased serotonin turnover rates [18] and changes of SERT activity [19] by LPS.

Acupuncture has been increasingly used as an alternative therapy for mental disorders such as addiction, Parkinson’s diseases, insomnia, and anxiety [20–23]. Especially, it was reported that acupuncture decreases tension, anxiety, and anger/aggression in anxiety disorder patients [23]. In addition, experimentally electroacupuncture regulates levels of T lymphocyte subsets in plasma and thymus in stress-induced anxiety rats [24]. Based on these studies, acupuncture may be effective in reducing anxiety, although the underlying mechanism is unclear.

To explore whether acupuncture can suppress anxiety-like behaviors by modulating SERT in the DRN, the present study examined the effects of acupuncture on LPS-induced anxiety-like behaviors and expressions of SERT in the DRN in rats.

## Methods

### Animals

Male Sprague-Dawley rats weighing 270–300 g (Daehan Animal, Seoul, Korea) were housed in groups of 2–3 rats per cage in controlled temperature (23 ± 2 °C) and humidity (50 ± 10%) on a 12 h light-dark cycle (lights on at 8:00 am) with ad libitum food and water. All experimental procedures were approved by the Institutional Animal Care and Use Committees of Daegu Haany University and conducted in accordance with National Institutes of Health guidelines for the care and use of laboratory animals.

### Drug and chemicals

Lipopolysaccharide (LPS) and other chemicals were purchased from Sigma (Sigma, St. Louis, MO, USA). Primary antibodies for c-Fos (sc-52, Santa Cruz, CA, USA) and serotonin transporter (SERT; AB9726, Millipore, MA, USA) and donkey anti-rabbit Alexa Fluor 488 (A21206, Life Technologies, CA, USA) and 594 (A21207) were used for immunohistochemistry. LPS was dissolved in physiological saline and intraperitoneally (i.p.) administered at dose of 0.2 mg/kg.

### Acupuncture treatment

**Fig. 1:**
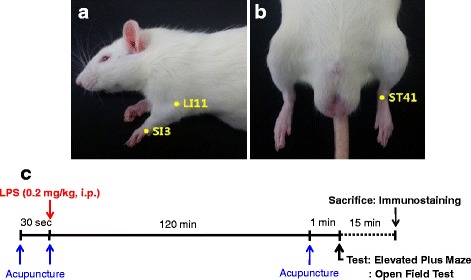
Acupuncture treatment and experimental procedure. **a, b** Location of SI3, LI11, and ST41 acupoints. **c** Experimental protocol. Rats were given three times acupuncture stimulation at SI3, LI11, and ST41 acupoint for 30 s before and 2 h after administration of lipopolysaccharide (LPS, 0.2 mg/kg, i.p)

Rats were given acupuncture at SI3, LI11, or ST41 acupoint for each 30 s before and after LPS administration (0.2 mg/kg, i.p.) and 2 h after LPS administration (Fig. 1a-c). The animals were then subjected to behavioral tests of elevated plus maze (EPM) and open field test (OPT) (Fig. 1c). For acupuncture treatment, stainless-steel needles (0.10 mm diameter and 7 mm length; Dongbang Medical Co., Korea) were inserted vertically to a depth of 3 mm from surface of skin, and manually performed twisted at a frequency of twice per second for 30 s, and the needles were then withdrawn. LI11 (*Qu Chi*) is located at the lateral of the transverse cubital crease midway, which is clinically prescribed for mental disorders in combination. SI3 (*Houxi*) is located on the dorsum of the hand, in the depression proximal to the ulnar side of the fifth metacarpophalangeal joint, at the border between the red and white flesh. ST41 (*Jiexi*) is located between two tendons on the dorsum of the foot which are more distinct when the ankle is dorsiflexion. In the present study, LI11 and SI3 acupoints were used as control points. The control groups were lightly grabbed without acupuncture needle insertion for 1 min.

### Elevated Plus Maze (EPM)

Anxiety-like behavior produced by LPS was measured by using a modified EPM method [25]. Briefly, the maze was constructed of black acrylic and consisted of two open arms (50 cm × 10 cm) and two closed arms (50 cm × 10 cm × 40 cm) extending from a central platform (5 cm × 5 cm). Rats were placed at the center of EPM and time spent in the open arms was recorded for 5 min by a video tracking system (Ethovision, Nodus Information Technology BV, Wageningen, Netherlands).

### Open field test (OFT)

A black rectangular box with a square floor (45 cm × 45 cm × 45 cm) was divided into nine equal sized zones (15 cm × 15 cm). Rats were placed at the central zone in an open field arena. Time spent was monitored and measured for 5 min in the central zone by a video tracking system (Ethovision, Nodus Information Technology BV, Wageningen, Netherlands).

### Immunofluorescence for SERT or c-Fos

Rats were anesthetized with sodium pentobarbital (80 mg/kg, i.p.) and intracardially perfused with ice-cold saline followed by ice-cold 4% paraformaldehyde solution in 0.1 M phosphate buffered saline (PBS; pH 7.4). Brains were rapidly removed from the skull and then post-fixed with 10% sucrose/4% paraformaldehyde for 2 h and cryoprotected in 30% sucrose for at least 48 h. The brains were cryosectioned into 30 μm slices and incubated in blocking solutions containing 0.3% Triton X-100, 5% normal donkey serum in 0.01 M PBS at the room temperature for 1 h. After rinsing in PBS, the sections were incubated with primary antibody for c-Fos (red; 1:1000, Santa Cruz, CA, USA) and serotonin transporter (green; 1:500, Millipore, MA, USA) overnight at 4 °C. The sections were then processed with secondary antibody with donkey anti-rabbit Alexa Fluor 594 (red; 1:500, Life Technologies, CA, USA) and 488 (green; 1:500). All sections were cover-slipped with a mounting medium (Vector Laboratories, Burlingame, CA, USA) and were imaged under a 10 X objective using microscope (Zeiss Axioskop, Oberkochen, Germany). Fluorescence intensities (FI) of SERT in each section were estimated by computerized densitometry (i-solution, IMT, Daejeon, Korea).

### Statistical analysis

Statistical analysis was carried out using SPSS 11.0 software. All data are presented as mean ± SEM (standard error of the mean) and were analyzed by one-way analysis of variance (ANOVA) followed by LSD post hoc test with statistical significance set at ^#^ *P* < 0.05, **, ^$$^ *P* < 0.01, and ***, ^###^ *P* < 0.001.

## Results

### Effect of acupuncture on EPM parameter after LPS administration

**Fig. 2:**
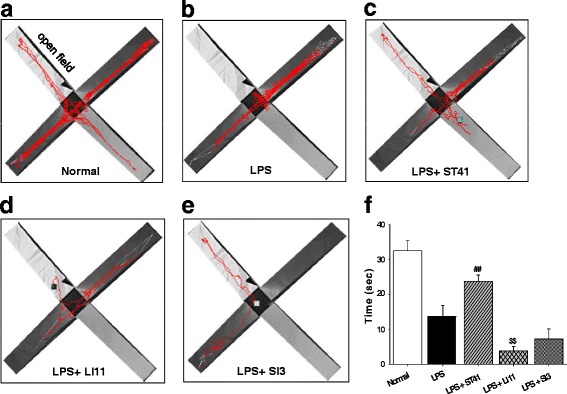
Effect of acupuncture on LPS-induced anxiety-like behavior in the EPM. **a-e** Representative examples of traveling pattern for 5 min in the open arms. **f** Time spent in the open arms for 5 min presented as mean ± SEM. While LPS-administered rats spent less time in open arms compared to normal, acupuncture at ST41, but neither LI11 nor SI3, significantly spent more time in open arms compared to LPS-administered rats. *** *P* < 0.001 vs. normal, ^##^ *P* < 0.01 vs. LPS, ^$$^ *P* < 0.01 vs. LPS; *n* = 6–7 per group

LPS-treated group significantly spent less time in the open arms compared to normal group (*P* < 0.001). As shown in Fig. 2b, the LPS-treated rats displayed avoidance of the open arms while staying in closed arms. In acupuncture groups, rats received acupuncture treatment of 3 sessions: before, immediately and 120 min after LPS administration (Fig. 1c). When time spent in the open arms was recorded for 5 min, acupuncture at ST41, but SI3, increased time spent in the open arms of EPM, compared to LPS-treated group (One-way ANOVA, *F*_(4,35)_ = 22.775, *P* < 0.001); post hoc, *P* < 0.01 vs. LPS). In contrast, acupuncture at LI11 (LPS + LI11) decreased time spent in the open arms of EPM compared to those of LPS or LPS + LI11 (Fig. 2; *P* < 0.01).

### Effect of acupuncture on OFT parameter after LPS administration

**Fig. 3:**
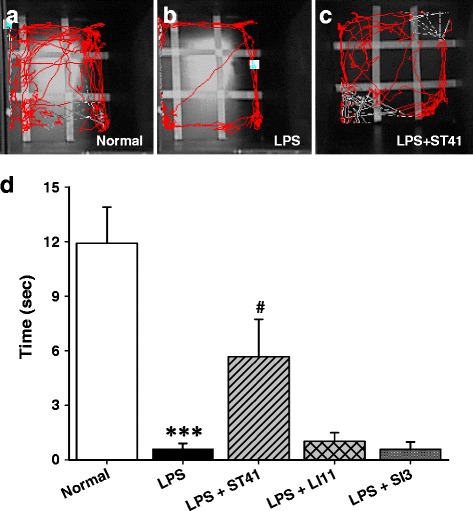
Effect of acupuncture on LPS-induced anxiety-like behavior in the OFT. **a-c** Representative examples of traveling pattern for 5 min in the open field. **d** Time spent in the center of zone for 5 min presented as mean ± SEM. While LPS-treated rats spent less time in the center zone of open field arena compared to control, acupuncture at ST41, but neither LI11 nor SI3, spent more time in center zone of open field arena compared to LPS-treated rats. *** *P* < 0.001 vs. control, ^#^ *P* < 0.05 vs. LPS; *n* = 6–7 per group

Since rodents tend to avoid the center of the field under stress or depressive condition [26], the OFT was performed to further confirm the effects of acupuncture at ST41 on anxiety-like behaviors. When arena was subdivided into center and border zones, LPS-treated group spent lesser time in the central zone of the open field area than control group (Fig. 3a and b, *P* < 0.001), indicating anxiety-like behaviors by EPM. On the other hand, acupuncture at ST41, but neither LI11 nor SI3, group significantly elevated time spent in the central zone of open field area (Fig. 3c and d; One-way ANOVA, *F*_(4,29)_ = 13.3707, *P* < 0.001; post hoc *P* < 0.05 vs. LPS).

### Effect of acupuncture on expression of SERT in the DRN

**Fig. 4:**
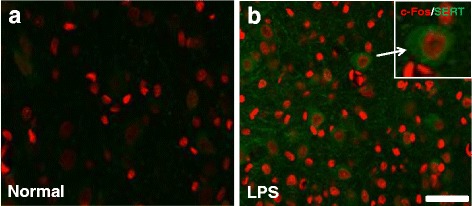
Expression of SERT in activated neurons in the DRN. **a, b** Representative images of expression of SERT (green) and c-Fos (red) in the DRN of normal (**a**) and LPS-treated rats (**b**). Enlarged image in (**b**) shows a DRN neuron double-labelled with SERT/c-Fos. Scale bar = 50 μm

To see whether SERT expression is increased in activated neurons after LPS administration, c-Fos, a marker of neuronal activation, was double-stained with SERT in the DRN. Many red stained nuclei (c-Fos) surrounded by green cytoplasmic staining were observed in LPS group, indicating the expression of SERT in activated DRN neurons (Fig. 4b).

**Fig. 5:**
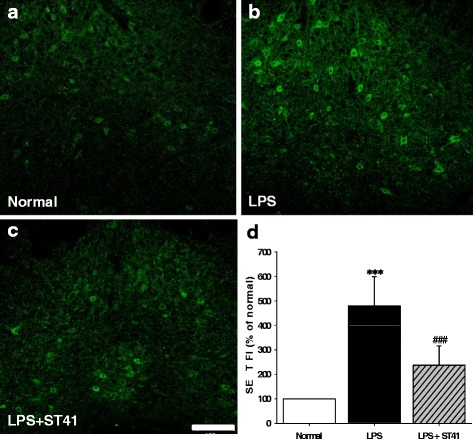
Effect of acupuncture on SERT expression in the DRN. **a-c** Representative images of SERT expression in the DRN. **d** Summary of SERT *fluorescence intensity* (FI, D) in the DRN following LPS administration. Data are expressed as percentage of control (normal). Acupuncture at ST41 markedly decreased SERT expression produced by LPS in the DRN. **P < 0.01 and ***P < 0.001 vs. control, ^###^ *P* < 0.001 vs. LPS, *n* = 3–4 per group. Scale bar = 50 μm

Next, to explore the changes of SERT expression in the dorsal raphe nuclei (DRN) following acupuncture at ST41 in LPS-treated rats, the brains were taken out 15 min after last acupuncture treatment in one set of rats, according to experimental procedure shown in Fig. 1c. An enhanced expression in SERT fluorescence was observed in LPS-treated group compared to normal rats (*P* < 0.01). Acupuncture at ST41 significantly attenuated the SERT expression compared to LPS group (Fig. 5. One-way ANOVA, *F*_(2,12)_ = 5.414, *P* = 0.021, post hoc *P* < 0.001 vs. LPS).

## Discussion

The present study demonstrated that acupuncture at ST41 results in 1) a decrease in LPS-induced anxiety-like behaviors in both EPM and OPF and 2) a reduction of SERT expression in the DRN enhanced by LPS.

LPS, a biologically active component of the outer membrane of gram negative bacteria, is widely used in experimental animal model in order to induce systemic inflammation [27], stimulate the release of pro-inflammatory cytokines in the brain areas [28] and produce sickness behaviors [29]. Peripheral LPS administration produces anxiety-like behaviors in EPM and OFT [30]. In accordance with others [31, 32], in the present study, LPS reduced time spent in open arms in EPM as well as in the center of zone in open filed area [33, 34], indicating induction of anxiety-like behaviors. These behaviors were reversed by acupuncture at ST41, but neither SI3 nor LI11. These results suggest that anxiety-like behaviors were induced by acute treatment with LPS and acupuncture could suppress the development of the anxiety in rats in a point-specific manner. Acupuncture at ST41 has been used empirically in conjugation with other acupoints to treat neurological disorders in humans, but few experimental studies have been conducted to support the effects of single point ST41 on neurological symptoms. In one previous study, acupuncture at ST41, without combination with other points, can generate therapeutic effect on muscle fatigue by reducing glutathione levels in muscle tissues [35]. It is first time to show experimental evidence of anxiolytic effects of ST41 acupoint.

Behavioral alterations in anxiety or depression disorder are closely linked to abnormalities of serotonergic system [36, 37]. As a large number of serotonin cells is found in the DRN [38, 39], the DRN (synthesis or releasing of serotonin) is considered to be a critical region related to anxiety or depressive disorder. Serotonergic neurons project from the DRN to the extended amygdala, hippocampus, striatum, nucleus accumbens, and cortex [40]. Several lines of evidence have shown that transport capacity (Vmax) of cortical SERT, SERT activity and SERT protein level are enhanced in the frontal cortex of LPS-administered animal [19] and in the DRN of chronic social defect animal model [41]. In addition, serotonin level is decreased in the DRN in stress-depressed rats [42]. In the present study, to observe the relationship between anxiety-like behavior and SERT in the DRN, the changes of SERT expression following LPS treatment were evaluated by immunohistochemistry. Our results showing that SERT expression was increased in the DRN in LPS-induced group may suggest that LPS-induced anxiety behaviors might be due to excessive reuptake of serotonin and in turn decreased level of serotonin in extrasynapse. Furthermore, in our present study, acupuncture at ST41, but not at control points (LI11 and SI3), significantly attenuated SERT expression in the DRN. These results indicate that acupuncture at ST41 could alleviate the anxiety-like behavior by suppressing SERT expression. According to other studies, acupuncture increases the serotonin level in the DRN [43] and the nucleus accumbens [44] and serotonin/5-hydroxyphenlyacetic acid (5-HIAA) ratio in the DRN [43]. These studies support that acupuncture may regulate serotonin system in the DRN and thus attenuates anxiety-like behaviors.

As the other possible mechanism, acupuncture may regulate the level of inflammatory cytokines produced by LPS. Peripheral administration of LPS induces inflammatory cytokines such as tumor necrosis factor (TNF) α, interleukin (IL)-1β, IL-6 [45, 46] which may produce depressive or anxiety-like behavior [14, 17, 47, 48]. Several lines of evidence have shown that acupuncture significantly decreases the level of proinflammatory cytokines in the brain areas in stress-induced depression model [49, 50]. Therefore, acupuncture may have an anxiolytic effect by reducing the level of pro-inflammatory cytokines in LPS-induced anxiety model. Further studies should be performed to confirm the mechanisms underlying the anti-inflammatory effect of acupuncture on LPS-induced anxiety model.

## Conclusions

In summary, acupuncture at ST41 acupuncture significantly can suppress LPS-induced anxiety-like behaviors in EPM and OFT and expression of SERT in the DRN produced by LPS. These results suggest that the anxiolytic-like effect of acupuncture may be achieved through regulation of SERT expression in the DRN.

## Abbreviations

DRN: Dorsal raphe nuclei
EPM: Elevated plus maze
LPS: Lipopolysaccharide
OFT: Open field test
SERT: Serotonin transporter
